# Modeling the impact of tuberculosis interventions on epidemiologic outcomes and health system costs

**DOI:** 10.1186/s12889-015-1480-4

**Published:** 2015-02-13

**Authors:** Olivia Oxlade, Amy Piatek, Cheri Vincent, Dick Menzies

**Affiliations:** McGill University and the McGill International TB Centre, Montreal, Canada; Montreal Chest Institute, 3650 St. Urbain St, Montréal, H2X 2P4 PQ Canada; United States Agency for International Development, Washington, DC USA

**Keywords:** Tuberculosis, Epidemiology, Decision analysis, Infectious disease modelling, Public health interventions

## Abstract

**Background:**

Tuberculosis (TB) programs must invest in a variety of TB specific activities in order to reach ambitious global targets. Uncertainty exists surrounding the potential impact of each of these activities. The objective of our study was to model different interventions and quantify their impact on epidemiologic outcomes and costs from the health system perspective.

**Methods:**

Decision analysis was used to define the TB patient trajectory within the health system of three different countries. We considered up to seven different interventions that could affect either the natural history of TB, or patient trajectories within the health system. The expected impact of interventions were derived from published studies where possible. Epidemiologic outcomes and associated health system costs were projected for each scenario.

**Results:**

With no specific intervention, TB related death rates are high and less than 10% of the population starts on correct treatment. Interventions that either prevent cases or affect all patients with TB disease early in their trajectory are expected to have the biggest impact, regardless of underlying epidemiologic characteristics of the setting. In settings with a private sector, improving diagnosis and appropriate treatment across all sectors is expected to have a major impact on outcomes.

**Conclusion:**

In all settings, the greatest benefit will come from early diagnosis of all forms of TB. Once this has been achieved more specific interventions, such as those targeting HIV, drug resistance or the private sector can be integrated to increase impact.

**Electronic supplementary material:**

The online version of this article (doi:10.1186/s12889-015-1480-4) contains supplementary material, which is available to authorized users.

## Background

Beginning in the mid-1980s, tuberculosis (TB) incidence dramatically increased globally, mostly attributed to urbanization in low and middle income countries (LMIC) and the HIV/AIDS epidemic. The emergence of multi drug-resistant TB (MDR-TB) has further contributed to the challenges of TB diagnosis and treatment. In response to the TB crisis, the World Health Organization (WHO) declared TB a global emergency in 1993. The Stop TB Partnership and the Global TB Drug Facility (GDF) [1,2] were created to help provide more support to countries, especially to National TB Programs (NTP), and by the late 1990s the United States Government started to provide funding to support NTP activities.

In 2006, WHO and the Stop TB Partnership launched an ambitious global plan to reach the Millennium Development Goal (MDG) targets of a 50% reduction in TB prevalence and mortality [3]. The Stop TB Strategy provided the operational plan to reach these targets and included a comprehensive approach to improve TB case detection and treatment outcomes [1]. Increases in funding by external donor agencies and some national governments have led to the implementation of the global plan and progress toward the MDGs [4].

As a result of these efforts, TB prevalence and mortality have declined. However, in 2013 there were 9.0 million new cases of TB and 1.5 million deaths from the disease [3]. A new post-2015 plan has been developed by global partners calling for increased investment in both evidence-based and innovative strategies to achieve 90% reduction in cases and zero TB deaths [5,6]. Given the uncertainty on how to effectively and efficiently achieve these ambitious objectives, we have modeled the potential impact of different TB interventions on long term epidemiologic outcomes and health system costs.

## Methods

### General approach to modeling

We used decision analysis to compare the impact on epidemiologic outcomes, and health system costs of different interventions to strengthen TB programs in LMICs that could affect either the natural history or patient trajectories of TB disease. Pathogenetic and epidemiologic inputs, as well as the impact of interventions, were derived from the published literature. The model predicted the number of new primary and associated secondary TB cases, TB mortality, and TB related national health system costs over 20 years (2001-2020).

#### Population

Three countries (Indonesia, Kazakhstan, and Mozambique) with different initial incidence of active disease, prevalence of drug resistance, HIV co-infection and health provider landscape were considered. Indonesia was considered representative of high TB burden, but low drug resistance and low HIV burden, with a private sector. Kazakhstan was considered representative of high TB burden, with high drug resistance, but low HIV burden and no private sector and Mozambique was considered high TB burden, with low drug resistance, and high HIV burden and with no private sector.

For each country-specific epidemiologic data relating to TB incidence, treatment outcomes, drug resistance and HIV co-infection were taken from published sources [7-9]. Drug resistance was categorized as multi drug resistant (MDR-TB) - which included any form of Rifampin resistance or drug sensitive (DS-TB). DS-TB included all cases that were not classified as MDR-TB, including those that were mono- and poly-drug resistant.

#### Overview of decision analysis model

A decision analysis model was constructed based on a conceptual framework developed to represent the natural history of TB and opportunities for intervention (see Additional file 1: Figure S1 for more detail). The population cohort was initially divided into those with and without TB infection. The population was then further stratified by HIV infection status as well as drug resistance.

Each year, those who were uninfected with TB could stay uninfected or acquire infection. Those with TB infection could remain without disease (with latent infection), or develop active disease in each year; this rate was higher in the first two years after infection. As summarized in Additional file 1: Figure S2 in the supplement, individuals with active TB could then begin seeking care. Individuals could either seek care without any delay, or after a delay. Depending on the setting, patients could seek care at different types of health facilities (in up to 3 sectors –public, informal (such as natural healers), or private). In each sector a correct or an incorrect diagnostic test could be ordered. If an incorrect test was ordered an individual would not be diagnosed, and would remain untreated. If a correct diagnostic test was ordered, this test could be ordered immediately, or with a delay. Some patients will not continue through the process to receive a diagnostic result. If diagnosed, the treatment prescribed could be correct (i.e. an NTP- or WHO-recommended regimen), or incorrect. If incorrect it was assumed the therapy was ineffective and cure rates were lower than those specified for recommended therapy. TB treatment could be initiated immediately, or after a delay. For those with MDR-TB disease, patients could receive a drug susceptibility test (DST) and be prescribed appropriate treatment, or not receive a DST and be given treatment for DS-TB (considered ineffective). Of those who started on correct therapy some completed treatment and were cured, others lost to follow-up, were not cured, relapsed or died. Active cases that received no treatment or inadequate treatment were considered to continue to transmit *M. tuberculosis* to the community until they either cured spontaneously or died from TB. This contributed to the number of secondary active cases generated during the simulation. Cases that incurred any delay were considered to continue to transmit the bacilli and could die during the period of delay. Specific probabilities of all of these events occurring are described below.

### Pre-intervention inputs

#### Epidemiologic inputs

Epidemiologic inputs (initial annual risk of TB infection, HIV and drug resistance rates) varied by country. Pathogenetic inputs and outcomes for undiagnosed/untreated cases varied by HIV status. Input values, taken from published studies, are summarized in the supplemental appendix (Additional file 1: Table S1).

#### Diagnostic and treatment related inputs

Pre-intervention diagnostic variables are summarized in Additional file 1: Table S2 and S3 in the supplemental appendix. Most inputs varied by sector (where relevant), but not by country, and were identified through an extensive systematic review of published studies of the effect of programme interventions on TB diagnostic and treatment outcomes. Since published estimates of patient, diagnostic, and treatment delays are generally average times in days or months, these average delays were converted into the probability of a one year delay. For example, an average delay of 30 days was considered equivalent to a one year delay for 8% of those with active TB seeking care, and no delay for the remaining 92%. TB treatment outcomes varied by country, type of underlying drug resistance, HIV status, and if DST was performed (Additional file 1: Table S3 in the supplemental appendix). In the pre-intervention scenario, HIV infected individuals were assumed to have no access to anti-retroviral therapy (ART).

#### Health system costs

All costs are summarized in Additional file 1: Table S4 in the supplemental appendix. Per item health system costs included those associated with TB diagnosis and treatment. TB treatment costs included drug costs, costs associated with DOT visits, and monthly medical follow up costs. Treatment costs were calculated separately for DS cases and MDR cases to reflect the higher drug costs and much longer duration of follow up. For DS or MDR cases that were lost to follow up, 50% of the full treatment cost was attributed. Medical visit costs (ie. medical follow up and treatment visit costs) varied by country [10,11] and were adjusted using World Bank data [12]. Diagnostic test, DST and drug costs were priced using WHO CHOICE data [13] and other international suppliers [2]. Costs associated with the implementation of the interventions were not included, because none of the published studies providing information on the impact of interventions considered provided corresponding cost estimates. All costs are in 2010 US Dollars.

### Interventions

Using the conceptual framework described above (see Additional file 1: Figure S1 for more detail), TB related interventions were matched to the stages of a TB patient’s natural history and trajectory through the health system. For example, investment in laboratory strengthening was considered an intervention that would affect diagnosis during the “diseased” stage of the framework. Interventions were considered if they had been supported by external funding agencies in the past and were prioritized during periods of NTP strengthening or expansion since 2000. Interventions were grouped into one of seven categories: 1) Community education, 2) Expansion of TB diagnostic network (DOTS expansion for Diagnosis), 3) Education and supervision of health care workers about correct treatment regimens (DOTS expansion for treatment), 4) Other DOTS expansion interventions not specifically related to diagnosis or treatment (Non Specific DOTS expansion- NTP strengthening), 5) Private sector interventions, 6) Expanded access to DST and reduced loss to follow up during treatment – for MDR (MDR-TB related Interventions), 7) Expanded access to ART for HIV co-infection (HIV/ART Therapy Programs). Each category of intervention could involve several potential specific activities. For example, for Intervention 3 (DOTS expansion for treatment) activities could include training of doctors, nurses and pharmacists on TB guidelines, monitoring and management of supplies of high quality drugs or translation and printing of training materials for community based DOTS. However, we assumed that these different activities would result in similar impacts in the model, and did not model the impact of these specific activities separately.

#### Key model parameters affected by intervention

(Table 1) For each intervention one or more model parameters were assumed to change over time following the intervention. For example for Intervention 2 (DOTS expansion for diagnosis) three different model probabilities were assumed to change (probability of incorrect diagnostic test ordered, diagnostic delay and loss to follow up during diagnostic work up). The interventions, together with corresponding probabilities assumed to change with each intervention, are summarized in Table 1. Many parameters relating to the effect of types of interventions were obtained through a systematic review of published studies of the impact of TB control interventions on TB outcomes and indicators, and were assumed to be the same in all 3 countries. A few pre-intervention parameters varied by country (e.g., frequency of loss to follow up), but most were assumed to be the same in all three countries.

**Table 1:** **Pre and post intervention values for specific model parameters**

**Intervention (all public sector unless otherwise specified)**	**Model parameters influenced**	**Pre-intervention value**	**Notes and reference for pre intervention**	**Post-intervention value**	**Notes and Reference for post intervention**
Community Education	Patient delay (probability of seeking care with a 1 year delay)	41 · 79 days = 0 · 11 probability of a 1 year delay	[14-17]	21 days = 0 · 06 probability of a 1 year delay	Assume 50% reduction in delay days
DOTS expansion for diagnosis	Incorrect diagnostic test ordered by heath professional	0 · 603	[18]	0 · 351	[19]
	Diagnostic delay (probability of incurring a 1 year delay)	29 · 49 days = 0 · 081 probability of a 1 year delay	[14-17]	1 · 83 days = 0 · 005 probability of a 1 year delay	Used pre-intervention data and ratio of delay days "pre" and "post" intervention from [20] to obtain post-intervention estimate of delay days
	Loss to follow up during diagnostic work-up	0 · 254	[21-24] (Assume that loss to follow up is the same for regardless of provider)	0 · 140	Used pre-intervention data and ratio of outcomes "pre" and "post" intervention from [19] to obtain post-intervention estimated of loss to follow up
DOTS Expansion for Treatment	Incorrect treatment	0 · 791	[25]	0 · 129	[25] Scenario assumed that incorrect treatment was given regardless of DST availability
Non specific DOTS Expansion (NTP Strengthening)	Initial access- inaccessible provider (ie · probability that patient seeks care with alternative provider that is inaccessible to interventions)	0 · 055	[14,26-31]	0 · 025	Intervention assumed to have same impact as in private sector
Private Sector interventions	Incorrect diagnostic test ordered by private provider	0 · 622	[18]	0 · 362	[19]
	Diagnostic delay (private sector only)	0 · 11	[14-17] (# days pre-intervention)	0 · 007	Used pre-intervention data and ratio of delay days "pre" and "post" intervention from [20] to obtain post-intervention estimate of delay days and then used ratio of outcomes in public vs private sector from [14] [27,32,33] to extrapolate estimate for public system to private system
	Loss to follow up during diagnosis (private sector only)	0 · 254	[21-24]	0 · 140	Assumed to be same as in public sector (a 45% reduction). Used pre-intervention data and ratio of outcomes "pre" and "post" intervention from [19] to obtain post-intervention estimated of drop out
	Incorrect treatment by private provider	0 · 771	[34]	0 · 126	Used pre-intervention data and ratio of outcomes "pre" and "post" intervention from [25] to obtain post-intervention estimate of incorrect treatment
HIV/ ART therapy programmes	TB Death rate in HIV/TB co-infected	0 · 12	[35]	0 · 10	[35] [36,37] (see table S5 in Supplement appendix for more detail)
	TB Relapse rate HIV/TB co-infected	0 · 16	[36]	0 · 01	[35] [36,37] (see table S5 in Supplement appendix for more detail)
	TB Reactivation rate HIV/TB co-infected	0 · 0340	[38-40]	0 · 02	[41]
MDR-TB related interventions	DST performed	0 · 2	Assumption	0 · 5	Assumption
	MDR- loss to follow up rate in HIV negative cases	0 · 22	[36]	0 · 11	Assumption- reduce rate to 50%

### Projected outcomes

Projected outcomes, over a 20 year time frame, included: primary active cases, secondary active cases generated from primary cases, TB related deaths (during diagnosis or treatment phases), and health system costs (from the perspective of the national health system in the 3 countries).

To better understand the contribution of changing specific model parameters associated with general interventions, projected outcomes were presented separately for each model parameter assumed to be influenced by the intervention. Discounting was not used because a cost effectiveness analysis was not performed, and the primary predicted outcomes were epidemiologic.

### Sensitivity analysis

The individual effect of each key model parameter described in Table 1 was investigated in sensitivity analysis, by considering the impact of an absolute change of 25% for each parameter. In Indonesia, the sequential impact of implementing several interventions that target the public and private sector together was also considered. In Kazakhstan, the sequential impact of implementing several interventions that first strengthen the general health system, and then improve the diagnosis and treatment of MDR-TB was considered.

#### Ethics statement

This study used a hypothetical simulation model based on previously published data, so research ethics committee approval was not required.

#### Availability of supporting data

All supporting data used in models are provided in the main text and in accompanying supplementary files.

## Results

### Impact of interventions to improve diagnosis and treatment of TB in the public and/or private sectors in a low MDR/low HIV setting. Indonesia case study.

As shown in Table 2, under baseline conditions (no specific intervention) almost two-thirds of active TB cases are predicted to die, and cure rates are very low. This reflects the assumed problems affecting all stages of the patients’ trajectory in this baseline scenario, so that very few patients are diagnosed and treated correctly. As a result the number of secondary cases exceeds the reactivated primary cases – implying a net increase in incidence over time under this scenario. The impact of interventions that affect single parameters without changing other parameters, is predicted to be quite modest, as seen in Table 3. Interventions to improve diagnosis in the private and public sectors are predicted to result in the greatest reduction of deaths and secondary cases, while interventions to improve treatment in either sector will result in greatest improvement in cures but with less effect on deaths. Enhancing diagnosis will result in the greatest increase in health system costs, reflecting the costs of putting more people on treatment. Improvements in the diagnosis and treatment of MDR-TB, with greater performance of drug sensitivity testing and reduced default from treatment are predicted to have the least impact. This reflects that in the base case analysis, most patients are not diagnosed with TB at all, thus reducing substantially any possible benefit of improved diagnosis and treatment of MDR-TB (which requires that TB is first diagnosed). The sensitivity analysis showing the impact of changing each key parameter by an absolute value of 25% is shown in Additional file 1: Table S6.

**Table 2:** **Total projected TB related outcomes per 1,000 general population, in Indonesia over 20 years**

**Intervention**	**Specific parameter changed** ^**1**^			**Primary active cases arising in cohort over 20 years** ^**3**^	**Total projected outcomes related to the primary cases**			
	**Parameter**	**Pre-intervention**	**Post-intervention**		**Death during diagnosis/treatment phase**	**Cure due to treatment**	**Secondary cases generated from primary cases**	**Health system costs**
Baseline	-	-	-	19 · 27	12 · 52	0 · 97	28 · 87	$2,641 · 47
Community Education	Patient delay^2^	11%	6%	19 · 27	12 · 39	0 · 99	28 · 54	$2,696 · 70
DOTS expansion for diagnosis	Incorrect Diagnostic Test (in public sector)	60%	35%	19 · 27	11 · 90	1 · 24	27 · 38	$3,302 · 55
	Diagnostic Delay^2^ (in public sector)	8%	0 · 5%	19 · 27	12 · 48	0 · 98	28 · 71	$2,670 · 63
	Loss to follow up during Diagnosis (in public sector)	25%	14%	19 · 27	12 · 37	1 · 03	28 · 51	$2,800 · 83
DOTS Expansion for Treatment	Incorrect Treatment (in public sector)	79%	13%	19 · 27	12 · 02	2 · 33	27 · 89	$2,646 · 61
Non specific DOTS Expansion (NTP Strengthening)	Access Government Facility	43%	73%	19 · 27	12 · 42	0 · 99	28 · 62	$2,764 · 86
Private Sector interventions	Incorrect Diagnostic test (in private sector)	62%	36%	19 · 27	11 · 75	1 · 33	27 · 04	$3,455 · 53
	Diagnostic Delay^2^ (in private sector)	11%	0 · 7%	19 · 27	12 · 46	0 · 98	28 · 63	$2,685 · 49
	Loss to follow up during Diagnosis (in private sector)	25%	14%	19 · 27	12 · 35	1 · 05	28 · 46	$2,823 · 16
	Incorrect Treatment (in private sector)	77%	13%	19 · 27	11 · 97	2 · 48	27 · 79	$2,647 · 19
HIV/ ART therapy programmes	HIV (+) Death	12%	10%	19 · 27	12 · 52	0 · 97	28 · 87	$2,641 · 47
	HIV (+) Relapse	16%	1%	19 · 27	12 · 52	0 · 97	28 · 87	$2,641 · 47
	HIV (+) Reactivation	3 · 4%	2%	19 · 26	12 · 52	0 · 96	28 · 86	$2,640 · 66
MDR-TB related interventions	DST performed	20%	50%	19 · 27	12 · 52	0 · 97	28 · 87	$2,693 · 77
	Loss to follow up during MDR Treatment	22%	11%	19 · 27	12 · 52	0 · 97	28 · 87	$2,642 · 53

Notes: ^1^See Table 1 for more detail; ^2^Delay = % with 1 year delay; ^3^Primary cases are those which would arise from reactivation of pre-existing latent TB infection, or progression from newly acquired infection, but do NOT include cases arising from transmission from the primary cases.

(Change in estimate shown represents change relative to baseline for a change in only one parameter and all others remain at pre-intervention values).

**Table 3:** **Changes in projected TB related outcomes relative to baseline of no intervention, per 1,000 general population, in Indonesia over 20 years**

**Intervention**	**Specific parameter changed** ^**1**^			**Change in outcomes related to primary active cases and ranking of impact** ^**3**^							
	**Parameter**	**Pre-intervention**	**Post-intervention**	**Death during diagnosis and treatment phase**		**Cure due to treatment**		**Secondary cases generated from primary cases**		**Health system costs**	
				**Change in outcome**	**Rank of impact**	**Change in outcome**	**Rank of impact**	**Change in outcome**	**Rank of impact**	**Change in outcome**	**Rank of impact**
Baseline	-	-	-	12 · 52	-	0 · 97	-	28 · 87	-	$2,641 · 47	-
Community Education	Patient delay^2^	11%	6%	−0 · 14	7	0 · 02	7	−0 · 33	7	55 · 24	10
DOTS expansion for diagnosis	Incorrect Diagnostic Test (in public sector)	60%	35%	−0 · 62	2	0 · 27	4	−1 · 49	2	661 · 09	14
	Diagnostic Delay^2^ (in public sector)	8%	0 · 5%	−0 · 04	10	0 · 01	10	−0 · 16	10	29 · 16	7
	Loss to follow up during Diagnosis (in public sector)	25%	14%	−0 · 15	6	0 · 07	6	−0 · 36	6	159 · 37	12
DOTS Expansion for Treatment	Incorrect Treatment (in public sector)	79%	13%	−0 · 50	4	1 · 36	2	−0 · 98	4	5 · 15	5
Non specific NTP Strengthening	Access Government Facility	43%	73%	−0 · 10	8	0 · 02	7	−0 · 25	8	123 · 40	11
Private Sector interventions	Incorrect Diagnostic test (in private sector)	62%	36%	−0 · 77	1	0 · 37	3	−1 · 84	**1**	814 · 07	15
	Diagnostic Delay^2^ (in private sector)	11%	0 · 7%	−0 · 07	9	0 · 02	7	−0 · 24	9	44 · 02	8
	Loss to follow up during Diagnosis (private sector)	25%	14%	−0 · 17	5	0 · 08	5	−0 · 41	5	181 · 69	13
	Incorrect Treatment (in private sector)	77%	13%	−0 · 56	3	1 · 51	1	−1 · 08	3	5 · 72	6
HIV/ ART therapy programmes	HIV/TB Death rate	12%	10%	0 · 00	11	0 · 00	11	0 · 00	13	0 · 00	2
	HIV/TB Relapse rate	16%	1%	0 · 00	11	0 · 00	11	0 · 00	13	0 · 00	2
	HIV/TB Reactivation rate	3 · 4%	2%	0 · 00	11	0 · 00	11	−0 · 01	11	−0 · 81	**1**
MDR-TB related interventions	DST performed	20%	50%	0 · 00	11	0 · 00	11	−0 · 01	11	52 · 31	9
	Loss to follow up during MDR Treatment	22%	11%	0 · 00	11	0 · 00	11	0 · 00	13	1 · 07	4

Notes: ^1^See Table 1 for more detail; ^2^Delay = % with 1 year delay; ^3^Rank of Impact ranks the projected impact of each intervention on each outcome, relative to the baseline of no intervention.

(Change in estimate shown represents change relative to baseline for a change in only one parameter and all others remain at pre-intervention values).

**Figure 1:**
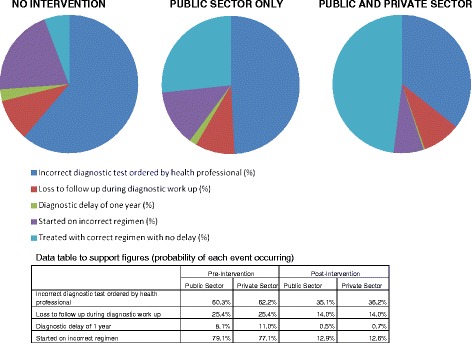
**Probability of intermediate outcomes if interventions are applied in public and or private sectors and achieve improvements in diagnosis and treatment as summarized below, in drug sensitive TB patients, in Indonesia.**

As shown in Figure 1, without any specific interventions, more than 60% of patients are lost to follow-up when the public or private provider orders an incorrect test, and another 25% are lost to follow-up prior to being diagnosed correctly. Of those diagnosed correctly, most are then placed on incorrect treatment; as a result less than 10% start on correct treatment. In Indonesia, where large numbers of TB patients access private providers, improvements only in the public sector will produce some benefits, but the greatest gains will be realized with a combined approach of interventions in both public and private sectors.

When the sequential addition of interventions in the public and private sector was assessed, improved diagnosis in all sectors would substantially reduce deaths and secondary cases, while improved treatment would cause further important reductions in these two outcomes (Table 4). A final reduction to near zero deaths and secondary cases would be achieved by eliminating all patients lost to follow-up prior to the initiation of treatment.

**Table 4:** **Projected outcomes over 20 years with progressive addition of interventions that improve the public and private sectors for TB care in Indonesia (per 1000 persons from general population)**

**Scenario**	**Primary active cases arising in cohort over 20 years** ^**1**^	**Total projected outcomes related to the primary active cases**				
		**Death during diagnosis phase**	**Death during treatment phase**	**Cure due to treatment**	**Secondary cases generated from primary cases**	**Health system costs**
Baseline	19 · 27	11 · 01	1 · 51	0 · 97	28 · 87	$2,641 · 47
Eliminate patient delay in seeking care	19 · 27	10 · 67	1 · 57	1 · 01	28 · 21	$2,751 · 94
Above & Eliminate informal sector for TB diagnosis	19 · 27	10 · 45	1 · 67	1 · 06	27 · 88	$2,916 · 34
Above & Increase correct diagnostic test to 100%	19 · 27	4 · 11	4 · 31	2 · 75	18 · 64	$6,809 · 05
Above & Eliminate health system diagnostic delay	19 · 27	3 · 67	4 · 44	2 · 84	17 · 40	$7,013 · 83
Above & Increase correct treatment to 100%	19 · 27	3 · 67	0 · 72	12 · 95	9 · 59	$7,052 · 06
Above & Eliminate loss to follow up prior to starting treatment	19 · 27	0	0 · 96	17 · 35	1 · 39	$9,295 · 09

^1^Primary cases are those which would arise from reactivation of pre-existing latent TB infection, or progression from newly acquired infection, but do NOT include cases arising from transmission from the primary cases.

### Impact of interventions to improve diagnosis and treatment of TB in a high MDR-TB or high HIV-TB setting. Kazakhstan and Mozambique case studies.

In Kazakhstan, using the pre-intervention scenario assumptions, a high death rate, low cure rate, and high number of secondary cases are predicted. As with Indonesia, interventions that change individual parameters one at a time will have modest effects (Additional file 1: Table S7). As summarized in Table 5, improved initial diagnosis and improved treatment of DS-TB (with first line drugs) are predicted to have the greatest impact on mortality, the number of secondary cases, and number of cases that are cured. The least impact on these three outcomes would result from isolated improvements in diagnosis of MDR-TB (increasing drug susceptibility testing from 20% to 50% of all DR cases), or improving MDR treatment (reducing loss to follow up from 22% to 11%), without any other programmatic changes.

**Table 5:** **Changes in projected TB related outcomes relative to baseline of no intervention per 1,000 general population, in Kazakhstan over 20 years**

**General intervention**	**Specific parameter changed** ^**1**^			**Projected changes in outcomes related to the primary active cases and Ranking of impact** ^**3**^							
	**Parameter**	**Pre-intervention**	**Post-intervention**	**Death during diagnosis and treatment phase**		**Cure due to treatment**		**Secondary cases generated from primary cases**		**Health system costs**	
				**Change in outcome**	**Rank of impact**	**Change in outcome**	**Rank of impact**	**Change in outcome**	**Rank of impact**	**Change in outcome**	**Rank of impact**
Baseline outcomes	-	-	-	10 · 05		0 · 62		22 · 99		$5,238 · 87	
Community Education	Patient delay^2^	11%	6%	−0 · 14	3	0 · 01	5	−0 · 26	5	111 · 34	3
DOTS expansion for diagnosis	Incorrect Diagnostic Test	60%	35%	−1 · 88	**1**	0 · 40	2	−2 · 47	**1**	2947 · 81	9
	Diagnostic Delay^2^	8%	0 · 5%	−0 · 11	4	0 · 02	4	−0 · 27	4	132 · 69	4
	Loss to follow up during Diagnosis	25%	14%	−0 · 45	2	0 · 10	3	−0 · 60	3	710 · 63	8
DOTS Expansion for Treatment	Incorrect Treatment	79%	13%	0 · 00	6	1 · 98	**1**	−1 · 54	2	363 · 20	7
Non specific DOTS Expansion (NTP Strengthening)	Access Government Facility	94 · 5%	97 · 5%	−0 · 09	5	0 · 02	4	−0 · 12	6	164 · 65	5
HIV/ ART therapy programmes	HIV/TB Death rate	12%	10%	0 · 00	6	0 · 00	6	0 · 00	8	0 · 00	2
	HIV/TB Relapse rate	16%	1%	0 · 00	6	0 · 00	6	0 · 00	8	0 · 00	2
	HIV/TB Reactivation rate	3 · 4%	2%	0 · 00	7	0 · 01	5	0 · 05	9	−142 · 43	**1**
MDR-TB related interventions	DST performed	20%	50%	0 · 00	6	0 · 02	4	−0 · 03	7	232 · 19	6
	Loss to follow up during MDR Treatment	22%	11%	0 · 00	6	0 · 00	6	0 · 00	8	10 · 11	3

Notes: ^1^See Table 1 for more detail; ^2^Delay = % with 1 year delay; ^3^Rank of Impact ranks the projected impact of each intervention on each outcome, relative to the baseline of no intervention.

(Change in estimate shown represents change relative to baseline for a change in only one parameter and all others remain at pre-intervention values).

This finding was explored further in sensitivity analysis summarized in Table 6. If the diagnosis and treatment of DS-TB were first improved - with 100% diagnosis and treatment and eliminating all patients lost to follow-up, but without changes in the MDR-TB program, the number of deaths from TB and secondary cases would fall by almost 70%, while the overall cure rate would increase from 4% to 75%. If the DR-TB program was also improved – by increasing DST coverage to 100%, and increasing treatment so that 100% received standard MDR-TB therapy, the overall cure rate would improve from 75% to 79%. If the MDR-TB regimen included new TB drugs that resulted in cure rates for MDR-TB equivalent to the cure rates now achieved for fully susceptible TB, this would result in an 8% further reduction in mortality, and an increase in overall cure rate to 84%.

**Table 6:** **Projected outcomes over 20 years with progressive addition of interventions that improve general TB services plus MDR diagnosis and treatment in Kazakhstan (per 1000 persons from general population)**

**Category of intervention**	**Scenario**	**Primary active cases arising in cohort over 20 years** ^**1**^	**Total projected outcomes related to the primary active cases**			
			**Death during diagnosis and treatment phase**	**Cure due to treatment**	**Secondary cases generated from primary cases**	**Health system costs**
	Baseline	15 · 28	10 · 05	0 · 62	22 · 99	$5,238 · 87
General Health System Interventions	Improved diagnosis to detect 100% of TB cases in public sector (DS and MDR)	15 · 28	7 · 67	1 · 57	17 · 07	$12,292 · 55
	Above & improved treatment to achieve 100% cure for DS in public sector	15 · 28	4 · 96	8 · 60	12 · 22	$12,354 · 14
	Above & reduced loss to follow up in public sector	15 · 28	2 · 70	11 · 53	7 · 23	$16,351 · 62
MDR-TB related interventions	Above & Improve DST coverage to 100%	15 · 28	2 · 59	11 · 72	6 · 96	$18,441 · 46
	Above & Improved treatment coverage so that all MDR cases diagnosed get standard MDR therapy	15 · 28	2 · 07	12 · 61	5 · 66	$27,327 · 96
	Above & New MDR drugs so treatment outcomes are as good as drug sensitive TB cases	15 · 28	1 · 85	13 · 11	5 · 41	$28,513 · 34

^1^Primary cases are those which would arise from reactivation of pre-existing latent TB infection, or progression from newly acquired infection, but do NOT include cases arising from transmission from the primary cases.

When these analyses were repeated using epidemiologic parameters from Mozambique (Tables 7 and 8), without any interventions, the number of deaths are much higher. Increasing ART treatment of HIV co-infected TB patients would result in the greatest reduction in mortality and secondary TB cases, plus produce net savings to the health system. This is the only intervention that results in a reduction of the number of primary TB cases, as it actually prevents TB cases. Interventions that enhance the laboratory network to improve diagnosis would have the next greatest impact.

**Table 7:** **Total projected TB related outcomes per 1,000 population, in Mozambique over 20 years**

**Interventions**	**Specific parameter change** ^**1**^			**Primary active cases arising in cohort over 20 years** ^**3**^	**Total projected outcomes related to the primary cases**			
	**Parameter**	**Pre**	**Post**		**Death during diagnosis and treatment phase**	**Cure due to treatment**	**Secondary cases generated from primary cases**	**Health system cost**
Baseline	-	-	-	69.82	47.34	2.40	106.05	$2,818.01
Community Education	Patient delay^2^	11%	6%	69.82	45.97	2.51	104.24	$2,955.56
DOTS expansion for diagnosis	Incorrect Diagnostic Test	60%	35%	69.82	39.57	3.92	94.82	$4,075.36
	Diagnostic Delay^2^	8%	0.5%	69.82	46.29	2.54	104.44	$2,942.14
	Loss to follow up during diagnosis	25%	14%	69.82	45.47	2.76	103.35	$3,121.12
DOTS Expansion for Treatment	Incorrect Treatment	79%	13%	69.82	47.34	10.00	99.21	$2,920.66
Non specific DOTS Expansion (NTP Strengthening)	Access Government Facility	94.5%	97.5%	69.82	46.96	2.47	105.50	$2,906.57
HIV/ ART therapy programmes	HIV/TB Death rate	12%	10%	69.82	47.34	2.45	106.05	$2,818.01
	HIV/TB Relapse rate	16%	1%	69.82	47.34	2.70	105.93	$2,818.01
	HIV/TB Reactivation rate	3.4%	2%	57.11	39.55	2.01	86.58	$2,326.66
MDR-TB related interventions	DST performed	20%	50%	69.82	47.34	2.41	106.02	$2,986.38
	Loss to follow up during MDR Treatment	22%	11%	69.82	47.34	2.40	106.05	$2,819.07

Notes: ^1^See Table 1 for more detail; ^2^Delay = % with 1 year delay; ^3^Primary cases are those which would arise from reactivation of pre-existing latent TB infection, or progression from newly acquired infection, but do NOT include cases arising from transmission from the primary cases.

(Change in estimate shown represents change relative to baseline for a change in only one parameter and all others remain at pre-intervention values).

**Table 8:** **Changes in projected TB related outcomes per 1,000 population, in Mozambique over 20 years**

**General intervention**	**Specific parameter changed** ^**1**^			**Projected changes in outcomes related to the primary active cases**			
	**Parameter**	**Pre**	**Post**	**Death during diagnosis and treatment phase**	**Cure due to treatment**	**Secondary cases generated from primary cases**	**Health system costs**
Baseline outcomes	-	-	-	47.34	2.40	106.05	$2,818.01
Community Education	Patient delay^2^	11%	6%	−1.37	0.11	−1.82	137.55
DOTS expansion for diagnosis	Incorrect Diagnostic Test	60%	35%	−7.77	1.52	−11.23	1257.35
	Diagnostic Delay^2^	8%	0.5%	−1.05	0.14	−1.61	124.13
	Drop out during Diagnosis	25%	14%	−1.87	0.37	−2.71	303.11
DOTS Expansion for Treatment	Incorrect Treatment	79%	13%	0.00	7.61	−6.84	102.65
Non specific DOTS Expansion (NTP Strengthening)	Access Government Facility	94.5%	97.5%	−0.38	0.08	−0.56	88.57
HIV/ ART therapy programmes	HIV/TB Death rate	12%	10%	0.00	0.05	0.00	0.00
	HIV/TB Relapse rate	16%	1%	0.00	0.30	−0.12	0.00
	HIV/TB Reactivation rate	3.4%	2%	−7.79	−0.39	−19.47	−491.35
MDR-TB related interventions	DST performed	20%	50%	0.00	0.02	−0.03	168.37
	Loss to follow up during MDR Treatment	22%	11%	0.00	0.00	0.00	1.06

Notes: ^1^See Table 1 for more detail; ^2^Delay = % with 1 year delay.

(Change in estimate shown represents change relative to baseline for a change in only one parameter and all others remain at pre-intervention values).

## Discussion

In our study, the greatest gains in reducing TB deaths and secondary cases are expected to come from interventions that reduce the reactivation rate to disease (ie. ART for HIV co-infected patients), or affect all patients with TB disease early in their trajectory, by improving their diagnosis. Further gains are made if treatment is also improved. In settings with a prominent private sector such as Indonesia, these achievements will be even greater if interventions are directed to both the public and private sectors. Even in settings with high levels of MDR-TB, we predict that interventions that improve diagnosis of all TB patients plus treat DS-TB cases correctly will have greater overall impact. The finding that the foundation for successful scale up of interventions is the strengthening of initial diagnosis and appropriate timely treatment of persons with TB supports current WHO recommendations for health system strengthening, TB prevention, diagnosis and treatment programs [42].

In the base case scenario, TB related mortality rates are projected to be very high in all settings, reflecting very low overall rates of successful diagnosis and treatment. In the model, TB cases that are undiagnosed die at rates consistent with those reported for smear positive cases in the pre-antibiotic era (approximately 33% per year [43]). In recent national prevalence surveys the number of TB cases that are undiagnosed has been found to be remarkably high. For example, in Nigeria, the case detection rate is now estimated to be only 16%, and TB mortality was found to be 400% higher than previously estimated rates [3]. Indonesia has also recently completed a prevalence survey but results are not yet published. In the 2014 Global TB report however, WHO suggests that results will lead to revisions of previously published global TB estimates [3].

The interventions included in this analysis are those most commonly introduced for TB prevention, diagnosis and treatment in LMIC as part of Stop TB Global plans, and were restricted to those for which there is published evidence of their potential impact. Several interventions were initially considered but ultimately excluded for the following reasons: 1) There was insufficient published data regarding their effect, 2) They had an overarching effect that influenced multiple elements within the conceptual framework so a precise effect within the model could not be assumed, or 3) published pre-intervention estimates were already excellent. The independent effects of each intervention can be very difficult to assess in field studies because in most countries multiple interventions have been applied simultaneously. Other modeling studies have considered the relative impact of different interventions [44-46], but not the impact of multiple interventions, nor multiple sectors. In this study we compared the relative impact of interventions separately, in combination, and across multiple sectors - which is more realistic. In addition, our study included health system costs which were not considered in detail in other studies which modeled epidemiologic outcomes [44-46].

Nevertheless this study has several limitations. First, although the number of secondary cases that originate from active cases were predicted in our model, these cases do not influence the annual risk of infection in subsequent years. Thus the population level impact of interventions was not directly evaluated and our findings are likely conservative. This limitation has implications particularly for DR-TB interventions, because preventing transmission (through prompt diagnosis and effective treatment) is an important goal. In all 3 countries modeled, interventions directed at improved diagnosis or treatment of MDR-TB were projected to have less impact than interventions to prevent primary cases, or to enhance early diagnosis of all TB cases. This is because patients with MDR-TB must first be diagnosed with TB, in order to have DST performed. Hence performing DST on a small fraction of all cases (when all the remaining cases have not been diagnosed at all) will inevitably have less impact than if diagnosis of all cases is improved first, since MDR-TB cases can only be diagnosed with DST. We also found that improving treatment of DS-TB cases would have more impact than improving treatment of MDR-TB, even when we assumed very high cure rates – similar to recently reported cure rates with new shorter MDR regimens [47]. This reflects the fact that even in a high MDR prevalence setting the majority of cases (>70%) are DS-TB. This is also combined with the fact that DS-TB regimens have a greater treatment success rate (averaging >90% [48]) than do MDR-TB regimens (averaging 55-60% [36]). As detection of DS-TB continues to improve, the impact of MDR-TB interventions will also have the potential to improve.

Second, the magnitude of the effect sizes for some of the interventions were large. Studies that documented the effect of interventions were identified through an extensive review of the literature, but some of these improvements were reported from single studies, some of which involved small study populations, or had short periods of follow-up. It is unclear if these improvements could be obtained or sustained when applied on a national scale. Finally, we could not perform a comprehensive cost effectiveness study, because the costs associated with interventions were not available from the studies that reported outcomes resulting from these interventions. This emphasizes the need for systematic collection and reporting of cost data in future studies of similar interventions.

In the post 2015 era there has been a call to accelerate progress by building on national and global efforts that have already had an impact on TB indicators [42,49]. Our sensitivity analysis on TB mortality (Table 4) considered in detail the substantial effort that will be required to meet the WHO goal of zero TB deaths. Scale up and strengthening of interventions like those included in our analysis should help to accelerate progress toward these ambitious goals, however the rate at which gains can be made will depend on the both the ongoing political and financial commitment to combat TB at both a global and national level.

## Conclusion

In all settings, the greatest benefit will come from interventions that reduce reactivation to disease, or those that increase early diagnosis and improve treatment for DS-TB as well as DR-TB. Once this has been achieved more specific interventions, such as those targeting HIV, drug resistance or the private sector can be integrated to increase impact. The findings of this study may provide useful information to guide selection of TB interventions in different settings, particularly as programs begin to scale-up interventions in the private sector and financial schemes are developed and improved to address universal health coverage.

## Additional file

Additional file 1: Figure S1. Framework for the natural history of TB disease, and opportunities for intervention. **Figure S2.** Summary of optimal patient trajectory and sub-optimal alternatives where interventions may be applied. Supplement **Table S1.** Epidemiology, Pathogenesis and Natural History parameters. Supplement Methods **Table S2.** Pre-Intervention (Baseline) Diagnostic and Treatment Related Parameters. Supplement Methods **Table S3.** Pre-Intervention Treatment outcomes with and without Drug Susceptibility Testing (DST). Supplement Methods **Table S4.** Summary of TB related Health System costs (All Costs in 2010 US dollars). Supplement Methods **Table S5.** Impact of Antiretroviral Therapy (ART) on TB TREATMENT OUTCOMES for TB/HIV positive patients, with and without Drug sensitivity testing (DST). Supplement Results **Table S6.** Sensitivity Analysis- Indonesia, Absolute change of 25% for key variables by intervention. Each variable run at assumed value of 25% and then at 50% (all other variables as per baseline scenario). Supplement Results **Table S7.** Total Projected TB related outcomes per 1,000 population, in Kazakhstan over 20 years.

## Abbreviations

USAID: United States Agency for International Development
TB: Tuberculosis
LMIC: Low and Middle Income Countries
HIV: Human Immuno-deficiency Virus
AIDS: Acquired Immuno Deficiency Syndrome
MDR-TB: Multi-Drug Resistant Tuberculosis
WHO: World Health Organization
GDF: Global Drug Facility
NTP: National Tuberculosis Program
MDG: Millennium Development Goals
MDR-TB: Multi Drug Resistant Tuberculosis
DS-TB: Drug Sensitive Tuberculosis
DST: Drug Sensitivity Testing
ART: Anti-Retroviral Therapy
DOTS: Directly Observed Therapy Short-course
