# Supplementary material for: Modeling the impact of tuberculosis interventions on epidemiologic outcomes and health system costs
Source: BMC Public Health. 2015 Feb 13;15:141. doi: 10.1186/s12889-015-1480-4 (PMC4335678; doi:10.1186/s12889-015-1480-4)
Supplement: Additional file 1: Figure S1. — Framework for the natural history of TB disease, and opportunities for intervention. Figure S2. Summary of optimal patient trajectory and sub-optimal alternatives where interventions may be applied. Supplement Table S1. Epidemiology, Pathogenesis and Natural History parameters. Supplement Methods Table S2. Pre-Intervention (Baseline) Diagnostic and Treatment Related Parameters. Supplement Methods Table S3. Pre-Intervention Treatment outcomes with and without Drug Susceptibility Testing (DST). Supplement Methods Table S4. Summary of TB related Health System costs (All Costs in 2010 US dollars). Supplement Methods Table S5. Impact of Antiretroviral Therapy (ART) on TB TREATMENT OUTCOMES for TB/HIV positive patients, with and without Drug sensitivity testing (DST). Supplement Results Table S6. Sensitivity Analysis- Indonesia, Absolute change of 25% for key variables by intervention. Each variable run at assumed value of 25% and then at 50% (all other variables as per baseline scenario). Supplement Results Table S7. Total Projected TB related outcomes per 1,000 population, in Kazakhstan over 20 years. [file 12889_2015_1480_MOESM1_ESM.docx]

**SUPPLEMENTARY MATERIAL:**

**Supplemental Figures, Methods and Results Tables:**

**Supplemental Figure:**

**Figure S1. Framework for the natural history of TB disease, and opportunities for intervention**

**
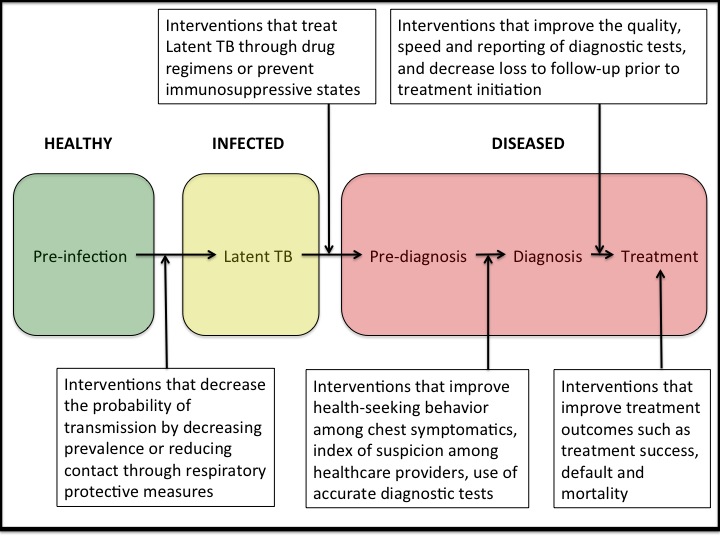
**

**UNINFECTED**

**Conceptual framework:** As shown in Figure S1, a framework was developed to represent the natural history of TB and the trajectories of patients with active TB disease. Three TB related states were identified: uninfected, infected (latent TB) and diseased (active TB). Opportunities for intervention to alter TB pathogenic parameters were identified between the “uninfected” and “infected” states as well as between the “infected” and “diseased” states. Within the “diseased” state three opportunities for intervention to alter patient trajectories existed: prior to diagnosis, at the time of diagnosis and during the treatment phase.

**Figure S2. Summary of optimal patient trajectory and sub-optimal alternatives where interventions may be applied**

**Description of Model:**

**Modeling Annual risk of TB infection:**

The model is a static decision analysis model. The annual risk of infection is calculated in the start year, using the Styblo formula (ARI= smear positive incidence/49) ([1](#_ENREF_1)), based on the World Health Organization estimated smear positive incidence rate in each country. The ARI is constant over the 20 year time frame, even in scenarios where interventions are in place.

Secondary cases are quantified as a projected outcome, using the assumption that 1 active case gives rise to 10 infections per year, with a 10% life time risk of progressing from infection to active TB (ie. 1 undiagnosed smear positive case leads to 1 secondary case per year), but these secondary cases do not directly influence the annual risk of infection in subsequent years, they are simplify quantified and provided as a model outcome.

**Modeling secondary cases as an outcome:**

Secondary cases are attributed each time there is a period of contagiousness due to an active case not being diagnosed, or left untreated. These secondary cases are attributed at each decision point where the transmission is assumed to occur , for example, during periods of diagnostic delay or if lost to follow up during treatment. Periods of contagiousness are additive, so that for example, if an individual incurs a one year delay at both the diagnostic stage, and the treatment stage, they will incur a total delay of 2 years. This delay is assumed to result in the development of 2 secondary cases which would contribute to the total number projected over the 20 year time frame. Periods of contagiousness that were used at different decision points in the model are summarized in Supplemental Table S2.

**Supplement Table S1: Epidemiology, Pathogenesis and Natural History parameters**

| Variable | | Value | Reference |
| --- | --- | --- | --- |
| EPIDEMIOLOGIC: | | | |
| Annual Risk of TB Infection (ARI) | Mozambique | 4.1%/yr | Calculated using Styblo formula ([1](#_ENREF_1)) and estimated smear positive incidence from around year 2000 ([2](#_ENREF_2)) |
|  | Kazakhstan | 1.3%/yr |  |
|  | Indonesia | 1.9%/yr |  |
| Prevalence of HIV | Mozambique | 11.3% | ([3](#_ENREF_3)) |
|  | Kazakhstan | 0.1% |  |
|  | Indonesia | 0.001% |  |
| Prevalence of initial MDR | Mozambique | 3.5% | ([4](#_ENREF_4)) |
|  | Kazakhstan | 14.2% |  |
|  | Indonesia | 2.0% |  |
| TB PATHOGENESIS: | | | |
| Probability of reactivation from longstanding infection (HIV Negative) | | 0.1%/yr | ([5](#_ENREF_5)) ([6](#_ENREF_6)) |
| Probability of reactivation from longstanding infection (HIV Positive) | | 3.4%/yr | ([7-9](#_ENREF_7)) |
| Probability of progressing to active TB (HIV negative) | | 5.0% (total) | ([10](#_ENREF_10), [11](#_ENREF_11)) |
| Probability of progressing to active TB (HIV positive) | | 33.0% (total) | Extrapolated based on ratio of HIV +/- reactivation rate |
| NATURAL HISTORY OF ACTIVE TB: | | | |
| Probability of spontaneously cure | | 25% (total) | ([12](#_ENREF_12)) |
| Probability of dying from smear positive TB if not diagnosed (HIV negative) | | 33%/yr | ([13](#_ENREF_13)) |
| Probability of dying if not diagnosed (HIV positive) | | 100%/yr | Assumption |

**Supplement Methods Table S2: Pre-Intervention (Baseline) Diagnostic and Treatment Related Parameters**

| Variable | Value | Reference |
| --- | --- | --- |
| PATIENT DELAY : | | |
| Probability of patient having a 1 year delay in seeking care for active TB | ( Mean Patient Delay in days/365 )= 11.5% | See “Mean delay“ section below for values in days |
| PATIENT CARE LOCATION: | | |
| Probability patient seeks care with private provider | Indonesia: 51.7% | ([14-20](#_ENREF_14)) |
|  | Kazakhstan and Mozambique: 0% |  |
| Probability patient seeks care with alternative provider that is inaccessible to interventions (eg. natural health care provider) | All settings: 5.5% |  |
| Probability patient seeks care in public health facility | Indonesia:42.8% | Complement to sum cohort to 100% |
|  | Kazakhstan and Mozambique: 94.5% |  |
| DELAYED DIAGNOSIS: | | |
| Probability of getting diagnosed with a 1 year delay (public sector) | ( Mean Diagnostic Delay in public system in days/365) = 8.1% | See “Mean delay ” below for values in days |
| Probability of getting diagnosed with a 1 year delay (private sector) | (Diagnostic Delay in public system)*1.31=10.6% | Refs for 1.31:([14](#_ENREF_14), [16](#_ENREF_16), [21](#_ENREF_21), [22](#_ENREF_22)) |
| LOSS TO FOLLOW UP: | | |
| Probability of loss to follow up prior to receiving correct diagnosis  ( public sector) | 25.4 % | ([23-26](#_ENREF_23))  (Assume that patient loss to follow up same for all providers) |
| Probability of loss to follow up prior to receiving correct diagnosis (private sector) |  |  |
| INCORRECT DIAGNOSTIC TEST: | | |
| Probability of ordering an incorrect diagnostic test ( public sector) | 60.3% | ([27](#_ENREF_27)) |
| Probability of ordering an incorrect diagnostic test (private sector) | 62.2% |  |
| DRUG SUCEPTIBLILITY TESTING (DST): | | |
| Probability of new cases receiving DST testing during diagnosis | 20% | Assumption |
| DELAYED TREATMENT: | | |
| Probability of starting treatment regimen with delay of 1 year (public sector) | ( Mean Treatment Delay in public system (days))/365 (days/yr)=0.8% | See “Mean delay” for values in days |
| Probability of starting treatment regimen with delay of 1 year (private sector) | (Treatment Delay in public system)*1.31=1.0% | Refs for 1.31: ([14](#_ENREF_14), [16](#_ENREF_16), [21](#_ENREF_21), [22](#_ENREF_22)) |
| INCORRECT REGIMEN: | | |
| Probability of starting an incorrect treatment regimen (public sector) | 79.1% | ([28](#_ENREF_28)) |
| Probability of starting an incorrect treatment regimen (private sector) | 77.1% | ([29](#_ENREF_29)) |
| Probability of using incorrect treatment regimen for MDR cases (no DST) | 100% | Assumption |
| CURE (after incorrect regimen) | | |
| Probability of cure after an incorrect regimen (non-MDR) | 62.4% | ([30-33](#_ENREF_30)) |
| Probability of cure after incorrect treatment for MDRTB | 44.0% | Assume same amount of difference as between cure after correct treatment for regular TB and incorrect treatment. (Incorrect Treatment therefore results in 71% less cure) |
| Probability of cure for after loss to follow up from correct treatment regimen | 62.4% | ([30-33](#_ENREF_30)) |
| MEAN DELAY (days): | | |
| Patient delay | 41.79 days | ([14](#_ENREF_14)) ([34-36](#_ENREF_34)) |
| Diagnostic delay | 29.49 days |  |
| Delay prior to receiving treatment | 2.9 days |  |
| PERIODS OF CONTAGIOUSNESS (for those who start treatment but die) | | |
| Between correct treatment regimen and death | 0.17 years | Assumption |
| Between incorrect regimen and death | 0.167 years | Assumption |
| Between loss to follow up during correct treatment and death | 0.17 years | Assume same as for death during correct treatment. |
| Between fail/relapse during correct treatment regimen and death | 0.417 years | Assumption |
| PERIODS OF CONTAGIOUSNESS (for those who are never treated) | | |
| Between developing disease and spontaneous cure | 1 year | Assumption |
| Between developing disease and death | 2 years | ([13](#_ENREF_13)) |

**Supplement Methods Table S3: Pre-Intervention Treatment outcomes with and without Drug Susceptibility Testing (DST)**

| Treatment Outcome by Country | Incorrect treatment for DS cases.  For MDR – no DST so treatment completely ineffective. | | | | With correct treatment (Assumes DST performed for MDR cases) | | | |
| --- | --- | --- | --- | --- | --- | --- | --- | --- |
|  | No HIV | | HIV** | | No HIV | | HIV** | |
|  | DS (Fully Susceptible) | MDR | DS (Fully Susceptible) | MDR | DS (Fully Susceptible) | MDR | DS (Fully Susceptible) | MDR |
| DEATH | | | | | | | | |
| Indonesia | 2.02% ([2](#_ENREF_2)) | 75%^Ϯ^ | 12.2% ([37](#_ENREF_37)) | 100%  (assumption) | 2.02% ([2](#_ENREF_2)) | 16% ([38](#_ENREF_38)) | 12.2% ([37](#_ENREF_37)) | 50% ([38](#_ENREF_38)) |
| Kazakhstan | 7.36% ([2](#_ENREF_2))* |  |  | 100%  (assumption) | 7.36% ([2](#_ENREF_2))* |  |  |  |
| Mozambique | 12.7% ([2](#_ENREF_2)) |  |  | 100%  (assumption) | 12.7% ([2](#_ENREF_2)) |  |  |  |
| FAIL/ RELAPSE | | | | | | | | |
| Indonesia | 1.25% ([2](#_ENREF_2)) | 0 | 16.2% ([37](#_ENREF_37)) | 0 | 1.25% ([2](#_ENREF_2)) | 8% ([38](#_ENREF_38)) | 16.2% ([37](#_ENREF_37)) | 4.76%^˄^ |
| Kazakhstan | 1.18% ([2](#_ENREF_2))* |  |  | 0 | 1.18% ([2](#_ENREF_2))* |  |  |  |
| Mozambique | 1.11% ([2](#_ENREF_2)) |  |  | 0 | 1.11% ([2](#_ENREF_2)) |  |  |  |
| LOSS TO FOLLOW UP | | | | | | | | |
| Indonesia | 4.95% ([2](#_ENREF_2)) | 0 | 4.95% ([2](#_ENREF_2))) | 0 | 4.95% ([2](#_ENREF_2)) | 22% ([38](#_ENREF_38)) | 4.95% ([2](#_ENREF_2)) | 13.1%^˄^ |
| Kazakhstan | 6.05% ([2](#_ENREF_2))* |  | 6.05%([2](#_ENREF_2)) * | 0 | 6.05% ([2](#_ENREF_2))* |  | 6.05% ([2](#_ENREF_2))* |  |
| Mozambique | 7.15% ([2](#_ENREF_2)) |  | 7.15% ([2](#_ENREF_2)) | 0 | 7.15% ([2](#_ENREF_2)) |  | 7.15% ([2](#_ENREF_2)) |  |
| CURE (Calculated as complement of (death + fail + relapse + loss to follow up) (except for spontaneous cure)) | | | | | | | | |
| Indonesia | 91.78% | 25%  (spontaneous  cure) ([12](#_ENREF_12)) | 66.65% | 0 | 91.78% | 54% | 66.65% | 32.1%^˄^ |
| Kazakhstan | 85.41% |  | 65.55% | 0 | 85.41% |  | 65.55% |  |
| Mozambique | 79.04% |  | 64.45% | 0 | 79.04% |  | 64.45% |  |

*No WHO data on treatment outcomes available for Kazakhstan in year 2000, so used average data from Indonesia and Mozambique

^Ϯ^  Mortality rate calculated as complement of spontaneous cure rate of 25%

**Assumes no ART available pre-intervention for HIV co-infected cases

^˄^ Death rate for MDR-TB HIV positive individuals obtained from literature ([38](#_ENREF_38)). Fail/Relapse and Cure rates for MDR-TB HIV positive individuals calculated to give same proportion of non fatal outcomes as reported for MDR-TB HIV negative individuals in ([38](#_ENREF_38)).

**Table S4. Summary of TB related Health System costs (All Costs in 2010 US dollars)**

|  | Cost | Reference/ Note |
| --- | --- | --- |
| DIAGNOSTIC HEALTH SYSTEM COSTS | | |
| Diagnostic test costs: | | |
| Cost of diagnostic test- liquid culture (per test) | $11.20 | ([39](#_ENREF_39)) |
| Cost of incorrect diagnostic test (per test) | $11.20 | Assumed to be same as correct diagnostic test |
| Cost of DST testing (per DST) | $20.00 | ([39](#_ENREF_39)) Cost includes procurement/distribution of lab supplies and reagents for DST. Does not include costs related to establishing a new DST lab, equipment, maintenance of transport of sample for diagnosis. |
| Medical visit costs: | | |
| Cost of a diagnostic/MD visit in Kazakhstan | $31.67 | ([40](#_ENREF_40)) ([41](#_ENREF_41))  Extrapolated to different countries using ([42](#_ENREF_42)) |
| Cost of a diagnostic/MD visit in Indonesia | $12.35 |  |
| Cost of a diagnostic/MD visit in Mozambique | $2.73 |  |
| TREATMENT RELATED HEALTH SYSTEM COSTS | | |
| Drug Costs: | | |
| Cost of a full regimen of standard First Line TB drugs (Drug susceptible TB) | $17 | ([43](#_ENREF_43)) |
| Cost of a full treatment regimen for MDR (assumes 8 Km Lfx Cs Eto Z / 16 Lfx Cs Eto Z) | $1,647 | ([43](#_ENREF_43)) |
| DOT costs: | | |
| Cost of a DOT visit in Kazakhstan | $10.54 | ([40](#_ENREF_40)) ([41](#_ENREF_41))  Extrapolated to different countries using ([42](#_ENREF_42)) |
| Cost of a DOT visit in Indonesia | $4.11 |  |
| Cost of a DOT visit in Mozambique | $0.91 |  |
| Sum of Diagnostic and Treatment Component Costs : | | |
| Total cost of first line drug treatment (if no DST performed, or for drug sensitive TB patients) | $1,134.54 Kazakhstan  $452.78 Indonesia  $113.46 Mozambique | Assumes 88 DOT visits, 6 follow up visits and a complete treatment regimen for drug sensitive disease |
| Total cost of MDR-TB treatment (for detected MDR TB patients) | $ 7887.88 Kazakhstan  $ 4080.60 Indonesia  $ 2185.72 Mozambique | Assumes 520 DOT visits, 24 follow up visits and a complete treatment regimen for drug resistant (MDR) disease |

**Table S5: Impact of Antiretroviral Therapy (ART) on TB TREATMENT OUTCOMES for TB/HIV positive patients, with and without Drug sensitivity testing (DST)**

|  | **Without Drug Sensitivity Testing (reference)** | | | | **With Drug Sensitivity Testing (reference)** | | | |
| --- | --- | --- | --- | --- | --- | --- | --- | --- |
|  | **Drug Sensitive- TB** | | **Drug Resistant-TB** | | **Drug Sensitive- TB** | | **Drug Resistant-TB** | |
| TB Treatment outcome | ART | No ART | ART | No ART | ART | No ART | ART** | No ART |
| DEATH | 9.7% ([37](#_ENREF_37)) | 12.2% ([37](#_ENREF_37)) | 75% * | 100% (Assumption) | 9.7% ([37](#_ENREF_37)) | 12.2% ([37](#_ENREF_37)) | 16% ([38](#_ENREF_38)) | 50% ([38](#_ENREF_38)) |
| FAIL/  RELAPSE | 1.1% ([37](#_ENREF_37)) | 16.2% ([37](#_ENREF_37)) | 0 | 0 | 1.1% ([37](#_ENREF_37)) | 16.2% ([37](#_ENREF_37)) | 8% ([38](#_ENREF_38)) | 4.76% ^˄^ |
| LOSS TO FOLLOW UP | 4.95% ([2](#_ENREF_2)) | 4.95% ([2](#_ENREF_2)) | 0 | 0 | 4.95% ([2](#_ENREF_2)) | 4.95% ([2](#_ENREF_2)) | 22% ([38](#_ENREF_38)) | 13.1%^˄^ |

Notes:

* Assumes 25% spontaneous cure, with 75% mortality – equivalent to non-HIV co-infected.

** Treatment outcomes for MDR-TB on ART assumed to be equivalent to MDR-TB HIV negative individuals (See table S3)

^˄^ Death rate for MDR-TB HIV positive individuals (no ART) obtained from literature ([38](#_ENREF_38)). Fail/Relapse and Cure rates for MDR-TB HIV positive individuals (No ART) calculated to give same proportion of non fatal outcomes as reported for MDR-TB HIV negative individuals in ([38](#_ENREF_38)) (See Table S3).

**Table S6: Sensitivity Analysis- Indonesia, Absolute change of 25% for key variables by intervention. Each variable run at assumed value of 25% and then at 50% (all other variables as per baseline scenario)**

| ***General Intervention*** | ***Specific Parameter Changed*** | | ***Projected Changes in Outcomes related to the primary active cases*** | | | |
| --- | --- | --- | --- | --- | --- | --- |
|  | **Parameter** | **Absolute change of 25%** | **Death during diagnosis and treatment phase** | **Cure due to treatment** | **Secondary cases generated from primary cases** | **Health System costs** |
| Baseline outcomes | - | - | 12.52 | 0.97 | 28.87 | $2,641.47 |
| Community Education | Patient delay | difference between 50% and 25% | -0.60 | 0.09 | -1.45 | 241.23 |
| DOTS expansion for diagnosis | Incorrect Diagnostic Test | difference between 50% and 25% | -0.62 | 0.27 | -1.48 | 655.84 |
|  | Diagnostic Delay | difference between 50% and 25% | -0.15 | 0.04 | -0.53 | 96.20 |
|  | Loss to follow up during Diagnosis | difference between 50% and 25% | -0.33 | 0.14 | -0.79 | 348.88 |
| DOTS Expansion for  Treatment | Incorrect Treatment | difference between 50% and 25% | -0.19 | 0.51 | -0.37 | 1.94 |
| Non specific DOTS Expansion (NTP Strengthening) | Access Government Facility | difference between 50% and 75% | -0.08 | 0.02 | -0.21 | 101.46 |
| Private Sector interventions | Incorrect Diagnostic test | difference between 50% and 25% | -0.75 | 0.35 | -1.77 | 784.27 |
|  | Diagnostic Delay | difference between 50% and 25% | -0.17 | 0.05 | -0.62 | 110.86 |
|  | Loss to follow up during Diagnosis | difference between 50% and 25% | -0.38 | 0.18 | -0.90 | 397.76 |
|  | Incorrect Treatment | difference between 50% and 25% | -0.22 | 0.59 | -0.42 | 2.21 |
| HIV/ ART therapy programmes | HIV/TB Death rate | difference between 50% and 25% | 0.00 | 0.00 | 0.00 | 0.00 |
|  | HIV/TB Relapse rate | difference between 50% and 25% | 0.00 | 0.00 | 0.00 | 0.00 |
|  | HIV/TB Reactivation rate | difference between 50% and 25% | 0.00 | 0.00 | 0.00 | -0.17 |
| MDR-TB related interventions | DST performed | difference between 50% and 75% | 0.00 | 0.00 | 0.00 | 43.59 |
|  | Loss to follow up during MDR Treatment | difference between 50% and 25% | 0.00 | 0.00 | 0.00 | 2.43 |

**Table S7: Total Projected TB related outcomes per 1,000 population, in Kazakhstan over 20 years.**

*(Change in estimate shown represents change relative to baseline for a change in only one parameter and all others remain at pre-intervention values****)***

| ***Interventions*** | ***Specific Parameter Change ^1^*** | | | **Primary Active cases arising in cohort over 20 years^3^** | ***Total Projected Outcomes related to the primary cases*** | | | |
| --- | --- | --- | --- | --- | --- | --- | --- | --- |
|  | **Parameter** | **Pre** | **Post** |  | **Death during diagnosis and treatment phase** | **Cure due to treatment** | **Secondary cases generated from primary cases** | **Health System costs** |
| Baseline | - | - | - | 15.28 | 10.05 | 0.62 | 22.99 | $5,238.87 |
| Community Education | Patient delay***^2^*** | 11% | 6% | 15.28 | 9.91 | 0.64 | 22.73 | $5,350.21 |
| DOTS expansion for diagnosis | Incorrect Diagnostic Test | 60% | 35% | 15.28 | 8.16 | 1.02 | 20.52 | $8,186.68 |
|  | Diagnostic Delay***^2^*** | 8% | 0.5% | 15.28 | 9.93 | 0.64 | 22.72 | $5,371.56 |
|  | Loss to follow up during Diagnosis | 25% | 14% | 15.28 | 9.59 | 0.72 | 22.39 | $5,949.50 |
| DOTS Expansion for  Treatment | Incorrect Treatment | 79% | 13% | 15.28 | 10.05 | 2.61 | 21.45 | $5,602.07 |
| Non specific DOTS Expansion (NTP Strengthening) | Access Government Facility | 94.5% | 97.5% | 15.28 | 9.95 | 0.64 | 22.87 | $5,403.52 |
| HIV/ ART therapy programmes | HIV/TB Death rate | 12% | 10% | 15.28 | 10.05 | 0.62 | 22.99 | $5,238.87 |
|  | HIV/TB Relapse rate | 16% | 1% | 15.28 | 10.05 | 0.63 | 22.99 | $5,238.87 |
|  | HIV/TB Reactivation rate | 3.4% | 2% | 15.23 | 10.05 | 0.63 | 23.04 | $5,096.44 |
| MDR-TB related interventions | DST performed | 20% | 50% | 15.28 | 10.05 | 0.65 | 22.96 | $5,471.06 |
|  | Loss to follow up during MDR Treatment | 22% | 11% | 15.28 | 10.05 | 0.63 | 22.99 | $5,248.98 |

Notes: ^1^ See methods table 3; ^2^ Delay = % with 1 year delay; ^3^Primary cases are those which would arise from reactivation of pre-existing latent TB infection, or progression from newly acquired infection, but do NOT include cases arising from transmission from the primary cases.

REFERENCES:

1. Styblo K. The relationship between the risk of tuberculous infection and the risk of developing infectious tuberculosis. Bull Int Union Tuberc Lung Dis. 1985;60(3-4):117-99.

2. World Health Organization. Global Tuberculosis Report 2011, Geneva. 2011.

3. HIV Global Database [apps.who.int/gho/data/]. Access date January 14th 2015

4. World Health Organization. Anti-tuberculosis drug resistance in the world: the WHO/IUATLD Global Project on Anti-Tuberculosis Drug Resistance Surveillance. Fourth global report. Geneva, Switzerland: WHO, 2008.

5. Comstock GW EL, Livesay VT. Tuberculosis morbidity in the US Navy: its distribution and decline. Am Rev Respir Dis 1974;110:572-80.

6. Nolan CM EA. Tuberculosis in a Cohort of Southeast Asian Refugees: A five-year surveillance study. Am Rev Respir Dis. 1988;137:805-9.

7. Wood R, Maartens G, Lombard CJ. Risk factors for developing tuberculosis in HIV-1-infected adults from communities with a low or very high incidence of tuberculosis. JAIDS-HAGERSTOWN MD-. 2000;23(1):75-80.

8. Whalen CC, Johnson JL, Okwera A, Hom DL, Huebner R, Mugyenyi P, et al. A trial of three regimens to prevent tuberculosis in Ugandan adults infected with the human immunodeficiency virus. New England Journal of Medicine. 1997;337(12):801-8.

9. Guelar A, Gatell JM, Verdejo J, Podzamczer D, Lozano L, Aznar E, et al. A prospective study of the risk of tuberculosis among HIV-infected patients. Aids. 1993;7(10):1345-50.

10. Grzybowski S BG, Styblo K. Contacts of cases of active pulmonary tuberculosis. Bull IUAT. 1975;50:90-106.

11. Sutherland I. The evolution of clinical tuberculosis in adolescents. Tubercle. 1966;47:308.

12. Grzybowski S ED. The fate of cases of pulmonary tuberculosis under various treatment programmes. Bull Int Union Tuberc 1978;53(2):70-4.

13. Rieder HL. Epidemiologic basis of tuberculosis control: International Union Against Tuberculosis and Lung Disease (IUATLD); 1999.

14. World Health Organization. Diagnostic and treatment delay in tuberculosis. Geneva: World Health Organization, , 2006.

15. USAID and TBCARE II. Study Report: Reducing TB Delays: Evaluating the Frequency and Causes of Delays in Bangladesh and Swaziland 2012.

16. Selvam JM, Wares F, Perumal M, Gopi P, Sudha G, Chandrasekaran V, et al. Health-seeking behaviour of new smear-positive TB patients under a DOTS programme in Tamil Nadu, India, 2003. The International Journal of Tuberculosis and Lung Disease. 2007;11(2):161-7.

17. Satyanarayana S, Nair SA, Chadha SS, Shivashankar R, Sharma G, Yadav S, et al. From where are tuberculosis patients accessing treatment in India? Results from a cross-sectional community based survey of 30 districts. PloS one. 2011;6(9):e24160.

18. Yamasaki-Nakagawa M, Ozasa K, Yamada N, Osuga K, Shimouchi A, Ishikawa N, et al. Gender difference in delays to diagnosis and health care seeking behaviour in a rural area of Nepal. The International Journal of Tuberculosis and Lung Disease. 2001;5(1):24-31.

19. Lienhardt C, Rowley J, Manneh K, Lahai G, Needham D, Milligan P, et al. Factors affecting time delay to treatment in a tuberculosis control programme in a sub-Saharan African country: the experience of The Gambia. The International Journal of Tuberculosis and Lung Disease. 2001;5(3):233-9.

20. Mahendradhata Y, Syahrizal BM, Utarini A. Delayed treatment of tuberculosis patients in rural areas of Yogyakarta province, Indonesia. BMC Public Health. 2008;8(1):393.

21. Rojpibulstit M, Kanjanakiritamrong J, Chongsuvivatwong V. Patient and health system delays in the diagnosis of tuberculosis in Southern Thailand after health care reform. The International Journal of Tuberculosis and Lung Disease. 2006;10(4):422-8.

22. Drabo KM, Dauby C, Coste T, Dembelé M, Hien C, Ouedraogo A, et al. Decentralising tuberculosis case management in two districts of Burkina Faso. The international journal of tuberculosis and lung disease. 2006;10(1):93-8.

23. Long Q, Li Y, Wang Y, Yue Y, Tang C, Tang S, et al. Barriers to accessing TB diagnosis for rural-to-urban migrants with chronic cough in Chongqing, China: a mixed methods study. BMC Health Services Research. 2008;8(1):202.

24. Krishnan A, Kapoor S. Involvement of private practitioners in tuberculosis control in Ballabgarh, Northern India. The International Journal of Tuberculosis and Lung Disease. 2006;10(3):264-9.

25. Khan MS, Khan S, Godfrey‐Faussett P. Default during TB diagnosis: quantifying the problem. Tropical Medicine & International Health. 2009;14(12):1437-41.

26. Dembele S, Ouédraogo H, Combary A, Sondo B, Macq J, Dujardin B. Are patients who present spontaneously with PTB symptoms to the health services in Burkina Faso well managed? The International Journal of Tuberculosis and Lung Disease. 2006;10(4):436-40.

27. Vandan N, Ali M, Prasad R, Kuroiwa C. Assessment of doctors' knowledge regarding tuberculosis management in Lucknow, India: A public–private sector comparison. Public health. 2009;123(7):484-9.

28. Indonesia TB Program Data 2006.

29. van der Werf MJ, Langendam MW, Huitric E, Manissero D. Knowledge of tuberculosis-treatment prescription of health workers: a systematic review. European Respiratory Journal. 2012;39(5):1248-55.

30. Chee C, Boudville I, Chan S, Zee Y, Wang Y. Patient and disease characteristics, and outcome of treatment defaulters from the Singapore TB Control Unit—a one-year retrospective survey. The International Journal of Tuberculosis and Lung Disease. 2000;4(6):496-503.

31. Parthasarathy R PR, Somasundaram PR.;. A controlled clinical trial of 3- and 5- month regimens in the treatment of sputum-positive pulmonary tuberculosis in South India. Am Rev Respir Dis. 1986;134:27-33.

32. East African/ British Medical Research Councils Study. Controlled clinical trial of five short-course (4-month) chemotherapy regimens in pulmonary tuberculosis: Second report of the 4th study. Am Rev Respir Dis. 1981;123:165-70.

33. Singapore Tuberculosis Service/British Medical Research Council. Long-term follow-up of a clinical trial of six-month and four-month regimens of chemotherapy in the treatment of pulmonary tuberculosis. Am Rev Respir Dis. 1986

(5):(133):779-83.

34. Storla DG, Yimer S, Bjune GA. A systematic review of delay in the diagnosis and treatment of tuberculosis. BMC public health. 2008;8(1):15.

35. Sreeramareddy CT, Panduru KV, Menten J, Van den Ende J. Time delays in diagnosis of pulmonary tuberculosis: a systematic review of literature. BMC infectious diseases. 2009;9(1):91.

36. Steffen R, Menzies D, Oxlade O, Pinto M, de Castro AZ, Monteiro P, et al. Patients' costs and cost-effectiveness of tuberculosis treatment in DOTS and non-DOTS facilities in Rio de Janeiro, Brazil. PLoS One. 2010;5(11):e14014.

37. Khan FA, Minion J, Pai M, Royce S, Burman W, Harries AD, et al. Treatment of active tuberculosis in HIV-coinfected patients: a systematic review and meta-analysis. Clinical Infectious Diseases. 2010;50(9):1288-99.

38. Ahuja SD, Ashkin D, Avendano M, Banerjee R, Bauer M, Bayona JN, et al. Multidrug resistant pulmonary tuberculosis treatment regimens and patient outcomes: an individual patient data meta-analysis of 9,153 patients. PLoS medicine. 2012;9(8):e1001300.

39. WHO CHOICE Costing tool. [http://www.who.int/choice/cost-effectiveness/inputs/en/.] Access date January 15th 2015

40. Law S, Benedetti A, Oxlade O, Schwartzman K, Menzies D. Comparing cost-effectiveness of standardised tuberculosis treatments given varying drug resistance. European Respiratory Journal. 2014;43(2):566-81.

41. Jacquet V, Morose W, Schwartzman K, Oxlade O, Barr G, Grimard F, et al. Impact of DOTS expansion on tuberculosis related outcomes and costs in Haiti. BMC Public Health. 2006;6(1):209.

42. The World Bank. www.worldbank.org. Access date January 14th 2015

43. Global Drug Facility. [http://www.stoptb.org/gdf/drugsupply/pc2.asp?CLevel=2&CParent=4.] Access date January 14th 2015
